# A Tropical Marine Microbial Natural Products Geobibliography as an Example of Desktop Exploration of Current Research Using Web Visualisation Tools

**DOI:** 10.3390/md20080028

**Published:** 2008-10-13

**Authors:** Joydeep Mukherjee, Lyndon E Llewellyn, Elizabeth A Evans-Illidge

**Affiliations:** 1 School of Environmental Studies, Jadavpur University, Kolkata 700 032, India E-mail: joydeep_envstu@school.jdvu.ac.in; 2 Australian Institute of Marine Science, Townsville, Queensland, Australia, 4810 E-Mail: E.Evansillidge@aims.gov.au

**Keywords:** tropics, marine, geospatial, biodiscovery

## Abstract

Microbial marine biodiscovery is a recent scientific endeavour developing at a time when information and other technologies are also undergoing great technical strides. Global visualisation of datasets is now becoming available to the world through powerful and readily available software such as Worldwind™, ArcGIS Explorer™ and Google Earth™. Overlaying custom information upon these tools is within the hands of every scientist and more and more scientific organisations are making data available that can also be integrated into these global visualisation tools. The integrated global view that these tools enable provides a powerful desktop exploration tool. Here we demonstrate the value of this approach to marine microbial biodiscovery by developing a geobibliography that incorporates citations on tropical and near-tropical marine microbial natural products research with Google Earth™ and additional ancillary global data sets. The tools and software used are all readily available and the reader is able to use and install the material described in this article.

**Technical primer for reader:** This paper consists of several parts. Prior to reading the paper, it is recommended that the reader undertake the following actions to enable visualization of global datasets as well as the tropical marine microbial bibliography described here.

1. If not already done, install Google Earth™ free version from http://earth.google.com/
2. Download file “Tropical marine microbial natural products geo bibliography.kml”
3. Download file “Supporting KML files marine microbial natural products geobibliography.kmz”
4. Start Google Earth™
5. Use File>Open in the menu and open the abovementioned files
6. A dataset list appears in a window on the right hand side of the screen enabling the reader to switch on or off the display of individual datasets
7. Some of the datasets require active internet connections to download the required information and the required data may take significant time to download during which time a small moving icon is often displayed by Google Earth™

## 1. Introduction

The use of biodiversity as a source of medicine is an ancient and well proven concept. At the start of the 21^st^ century, an estimated 75% of the world’s population continued to rely on traditional plant-based medicines for primary health care [102], and over 70% of new chemical entities developed since the 1940’s as new drugs for cancer treatment, were based on natural products [78]. However, global exploration for novel natural products did not seriously enter the ocean realm until relatively recently, prompted by the development and wide availability of modern, safe diving equipment in the 1960’s [18], and safe manned submersibles in the following decade [8]. Compared to the previous use of remote techniques such as dredging and trawling, this new technology enabled scientists to more selectively collect biodiversity, and provided the opportunity for natural history and other observations.

In its short history, marine natural products research has yielded over 15000 new chemical entities [10], and generated an exciting ‘marine pipeline’ of new drugs [94]. This productivity is not surprising, because marine habitats are the most biodiverse places on earth, harbouring taxa that are simply not accessible on land. Based on knowledge about higher taxonomic levels (phyla and classes), around 75% of known eukaryotic phyla occur in the sea, many of them exclusively [71]. An additional order of magnitude is superimposed by the immense biodiversity of marine microorganisms, which are often associated with macroorganisms through complex symbiotic and commensal relationships [77, 101, 106], but also populate seawater and sediment in environments that span the extremes of nutrients, pressure, salinity and temperature [28]. Even oligotrophic ocean waters are not the microbial desert that was once assumed [94]. The extent of marine microbial biodiversity, and consequently natural products potential, seems to be limitless and growing larger as new techniques emerge to measure it. Some marine natural products previously thought to have been produced by invertebrates, are now known to actually be microbial in origin [77].

Geographical information has an important role to play in the field of marine microbial natural products research. For example, demonstration of legal provenance for each source sample relies on a reliable link between the location sampled and the existence of appropriate permission for the collection and the subsequent use of material in biodiscovery endeavour, including the legal right to commercialise the outcomes. This is becoming increasingly important as a legal requirement of emerging domestic legislation in many countries, which is being enacted to implement the access and benefit sharing provisions of the Convention on Biological Diversity (CBD) [26]. International negotiations are currently underway to negotiate a new international regime for access and benefit sharing within the work-program of the CBD. Many developing countries are calling for this new regime to be legally binding with a requirement for proof of legal provenance [29, 110], and a proposal has been made by a consortia of countries to seek amendments to the WTO’s Trade Related aspects of Intellectual Property rights (TRIPS) agreement to support this by making disclosure and proof of legal provenance a mandatory prerequisite to the granting of Intellectual Property rights where innovation is based on the use of genetic resources [20, 34]. If this move is successful, an inability to demonstrate the source of genetic resources and associated CBD compliant permission, with reliable geo-spatial information, may invalidate a patent claim.

Geographical information about natural products, whether purified compounds, enzymes or organismal extracts, is also valuable when making strategic decisions about where to direct collection effort. If biogeographical data linked to taxonomy and ecological observations is accurately recorded when collecting samples for biodiscovery, then any resulting leads can be amplified in a number of ways. For example, one option is to examine material from other locations with similar biogeographical features, or target known stressors which may induce a wider variety of natural products. Another is to target environments known to yield natural products of interest due to metabolic specialisation such as in extreme environments [3]. Further, the suite of natural products produced by an organism often varies considerably with environmental parameters [65, 84] so leads can be elaborated by considering the same taxa from a variety of locations. The ability to data-mine appropriately curated relational databases and generate hypotheses for natural product discovery, can be optimised with accurate geo-spatial referencing and an effective visualisation tool.

There now exists a number of virtual tools including internet accessible databases and web visualisation tools which can be harnessed for natural products research. For example, Pubchem is a growing and freely available database provided by the National Center for Biotechnology Information of the US National Institutes of Health. Like Genbank which is a vehicle for the free exchange of genetic information and an enabler of its data mining, Pubchem includes small organic molecules and available bioactivity information linked to other freely available web-information such as publication information and toxicology databases. There are also range of accessible options for global visualisation, including Worldwind™ (NASA Ames Research Center, CA, USA), Google Earth™ (Google Incorporated, CA, USA) and ArcGIS Explorer™ (ESRI, CA, USA). These are freely available applications that enable visualisation of the globe’s surface in astonishing detail. The current study aims to bring these tools together to demonstrate desktop and geographically visualised exploration of global data – in this case, the global research effort in the field of marine microbial natural products, with a focus on research undertaken on culturable microorganisms sourced from tropical and near-tropical areas.

## 2. Materials and Methods

### Google Earth™

Google Earth™, was freely downloaded from http://earth.google.com/ and created the foundation for this project. Georeferenced data is read by Google Earth™ and similar applications using the Keyhole Markup Language (KML) format which can be written and edited using standard text editors. Thus, georeferenced data used in this project was compiled in KML format.

### Making the geobibliography

Almost 90 citations for marine microbiology culture-based natural products research in the tropics, defined as reported collection sites occurring between the Tropics of Cancer and Capricorn, were acquired from the following literature databases: PubMed (US National Library of Medicine, NIH, USA), Web of Knowledge^SM^ (ISI, Thomson Scientific, Philadelphia, USA) and Marinlit (ver 14.3, University of Canterbury, Canterbury, New Zealand). Only citations that satisfied the following criteria were included:

- the original microbial collection site was reported or could be determined from the article.
- the collection site was tropical or near-tropical
- the means of producing the natural product was primarily by culture of the micro-organisms rather than by simply collecting large wild populations (eg cyanobacterial mats or microscopic blooms)

A data record was then created for each compliant citation with the following fields:

- a name for the location;
- the citation for the work;
- a link to the publication’s digital object identifier (DOI), or in those few cases where a DOI was unavailable, directly to the article if it were freely available;
- a link to a PubChem [91] entry if it existed;
- longitude and latitude converted to decimal format. For those readers who examine the KML file with a text editor, note that the third number that appears after the longitude and latitude defines the height above ground level that Google Earth™ will display the icon for the placemark;
- a command that tells the earth visualization software where to find the icon image to use for each placemark. The icons used here are the flags of the country where the research organization to which the corresponding author belongs is located;
- the year of publication of the citation to enable the “time slider” function.

The resulting dataset [1, 2, 4, 5, 7, 9, 12, 13, 15-17, 19, 21–24, 27, 30–33, 35–37, 39–41, 43–58, 60–64, 66, 68–70, 72–76, 79–83, 85–90, 92, 93, 95–100, 104, 105, 107–109, 111, 112] was then compiled in KML format, and deployed as a data layer in Google Earth™.

### Ancillary data:

A range of other data sets in KML format were also acquired via free internet download, to assist in visualization based interpretation of the geobibliography. These data sets and their source, are summarized in Table 1.

## Results and Discussion

The geobibliography KML file is supplied with this article, and can be opened by the reader in any text editor or word processing software to view and edit the file structure. Alternatively, it can be installed as a data layer in Google Earth™ as described below.

### Instructions for installation and use of the tropical marine microbial natural product geobibliography supplied with this article

1. If not already done, install Google Earth™ free version from http://earth.google.com/
2. Download file “Tropical marine microbial natural products geo bibliography.kml”
3. Download file “Supporting KML files marine microbial natural products geobibliography.kmz” which is a compilation of the KML files containing the additional datasets listed in Table 1 and described in the ensuing text
4. Open Google Earth™
5. Use File>Open in the menu and open the abovementioned KML files
6. A list of the datasets will appear in a window on the right hand side of the screen and the display of the each dataset, and the elements within, can be switched on and off by clicking on their checkboxes
7. Note that some datasets require internet connections to interactively download required information and data download may take significant time. A small moving icon is often displayed by Google Earth™ to indicate that downloading is occurring.

A time slide in the upper right hand corner of the window, displays the time span of citations displayed (based on publication date). When the file first opens, by default it will only show those citations occurring in the early years. Clicking on the right hand side of the slider and dragging it to the far right will expand the time span and reveal the icons of all of the citations in the KML file. Similarly, the time slide can be adjusted by the user to restrict the citations displayed to a selected timeframe. Figure 12 gives an example of the time-differentiation of citations in the geobibliography for the Caribbean region. Once the required KML files are installed with Google Earth™, the reader can explore the datasets visually by empowering the full functionality of Google Earth™’s features.

**Figure 1.**
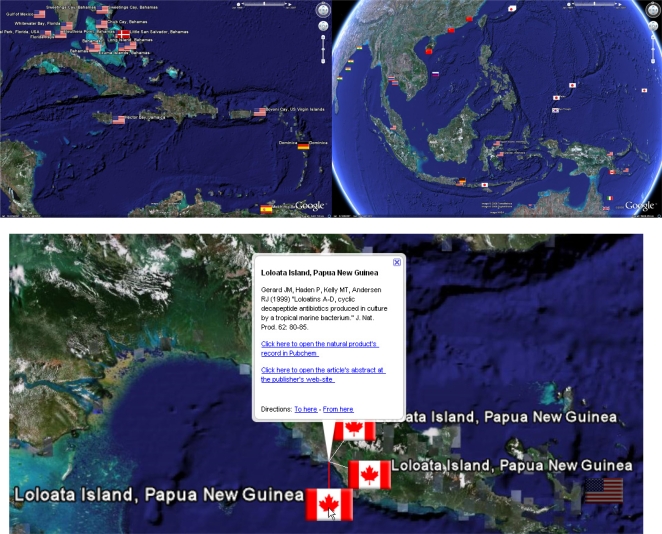
Images showing the basic features of the geobibliography. Panels A and B respectively show the Caribbean and part of the Asia-Pacific region. Panel C shows what the reader will see when they click on a placemark with the reference for the paper being shown as well as links to Pubchem and the publisher’s website for the reference in question.

Figure 1 shows a selection of example fields that illustrate the basic features of the geobibliography in Google Earth™, including an example citation record displayed by clicking the mouse on a placemark. The information box containing the citation record includes hyperlinks to the citation publisher’s web-site, from where the article may be accessed (pending on electronic subscriptions being in place). In some cases, as in the example in Figure 1 panel C, where the structure of the described compound (or a close chemical relative) can be found in Pubchem, a hyperlink to the Pubchem record is provided. Where more than one citation is co-located and their icons overlay each other, the cursor icon will change to a four-headed arrow indicating the presence of more than one icon. Clicking on the arrow will explode the collocated icons and allow selection of the individual icons.

Two important functions of Google Earth™ enable rapid browsing of the information contained within the geobibliography. Firstly, the search functionality in the “Fly To” window allows the user to insert the name of a geographic location and, if the search function finds it, navigate to that location. Alternatively, the user can also input the longitude and latitude values of a desired location and navigate to that location. For example, inserting “21N,156W” into the search box will take the user to Hawaii. Secondly, the “Places” panel usually located to left of the software window, can be used to navigate directly to the collection location for a citation. Clicking on the “Tropical marine microbial natural products bibliography.kml” in the Places panel will expand a list that shows each individual citation. Double-clicking on any of these entries will cause the software to go to the placemark on the globe’s surface.

The reliability of the location information used in the geobibliography varies with a number of factors. Firstly, while the cartographic system used in the Google Earth™ location is the WGS84 datum, many different datum systems have been used over the years and are typically not reported in the citations. Thus, where the position used in the citation has not used WGS84, it will be displayed slightly inaccurately when plotted onto Google Earth™. However, this discrepancy is unlikely to be significant. The difference between the various geodatums can be measured in seconds with each second converting to approximately 30 metres at the Equator but to shorter distances as the a location moves towards one of the Poles. Secondly, the precision of stated longitude and latitudes can also vary greatly depending on the method used to determine the position. For example, a position plotted on a paper chart using hand bearings will generally be less precise than a fix from a modern GPS receiver. Further, the latitude and longitude values reported in citations varied in the number of decimal places used, and therefore the precision of the fix.

It should be noted that the icon location in the geobibliography is where sample collections were undertaken. This is often different to the location where the research that is the subject of the citation took place. The reader should refer to the actual citation to obtain the reported geographical location and the associated precision and accuracy of the geographic location.

**Figure 2.**
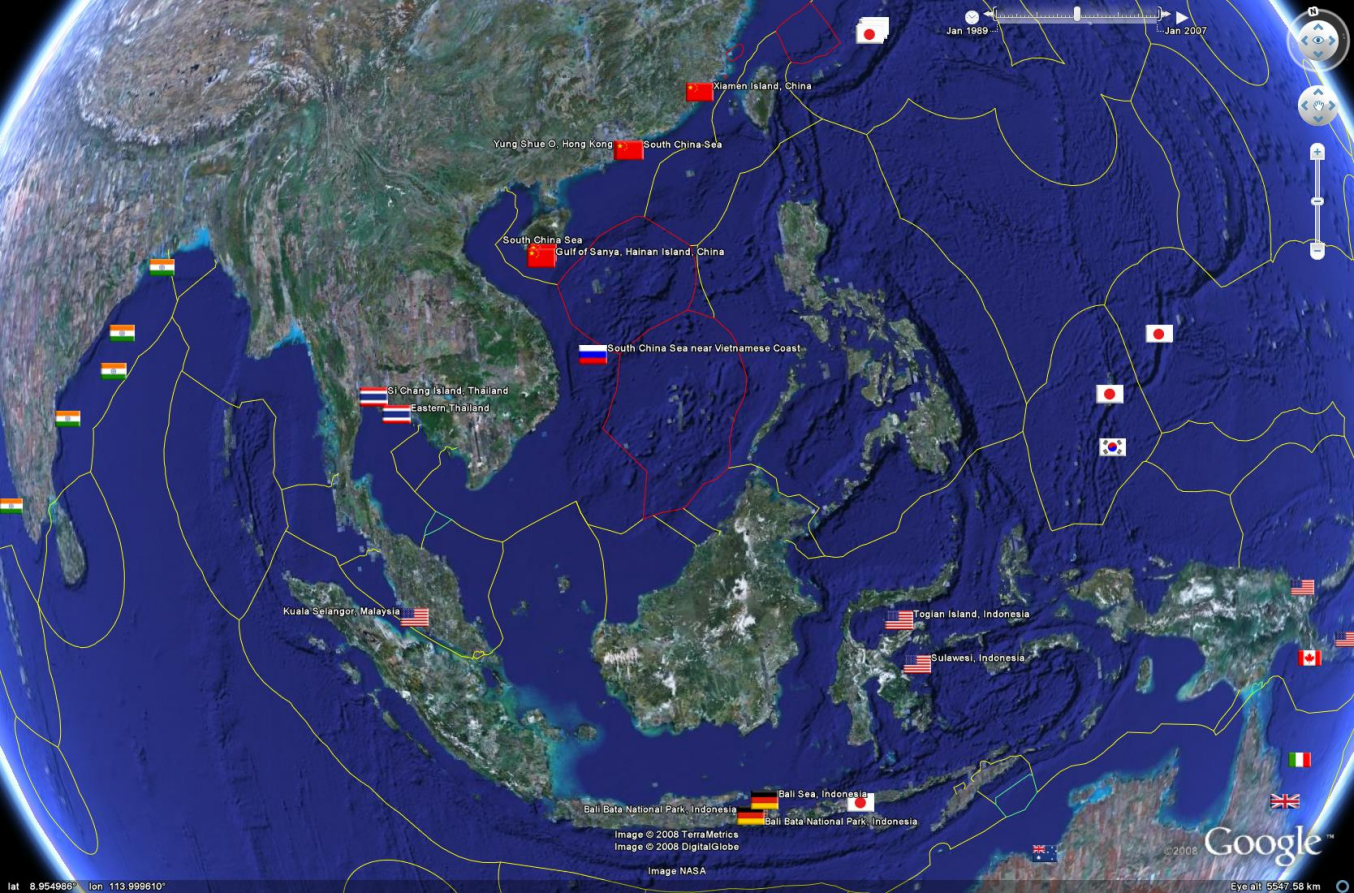
Two further views of the geobibliography along with the limits of Exclusive Economic Zones as calculated by the Vlaams Instituut voor de Zee. Note that the red borders are areas believed to be disputed

Potential inaccuracies introduced in location reporting in the citations should especially be considered when visually combining the geobibliography with KML file containing the boundaries of Exclusive Economic Zones (EEZ), as depicted in Figure 2. The boundaries used here include a subset of EEZ’s including some which are under dispute. The ability to visualise the location of collection effort with respect to areas of various national jurisdictions has great potential application for assertion of national sovereignty and monitoring access and benefit sharing, especially where a country wishes to be aware of research occurring within their borders. This must be done with caution however using the outputs of this present study, due to the potential inaccuracies of reported locations. Note also that some of the EEZ boundaries themselves are currently under dispute according to [6]. These boundaries are coloured red in Figure 2.

**Figure 3.**
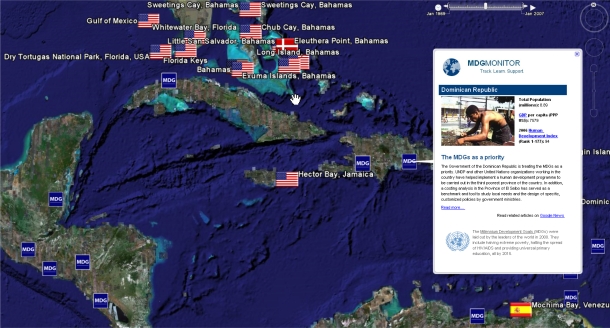
The Caribbean section of the geobibliography overlaid with the information from the Millennium Development Goal Monitor which provides socioeconomic information for every country in the world (http://www.mdgmonitor.org/).

Nevertheless, considering that each placemark in the geobibliography is the flag of the nation of the senior author, it is clear that microbial marine natural products research is a highly international endeavour with the flags of many nations occurring within the marine territories of other countries. In virtually all cases where this occurs, co-authors on the citation include scientists from the source country, indicating that cross-country collaborations that include the country of origin are common. Collaboration can provide an important avenue for some countries without the necessary skills, infrastructure and funds to undertake their own biodiscovery program. Many tropical regions and countries have very low Gross Domestic Products (GDP) [42], which is likely to limit resources available to undertake biodiscovery of any kind. Figure 3 visualises the Caribbean region with the geobibliography overlaid with information about Millennium Development Goals, including socioeconomic data such as GDP. The Millennium Development Goal project is an initiative of the United Nations to focus international efforts on global development standards [103]. Particularly, for some biodiverse but developing nations, collaborations have the potential to provide capacity building including skills development and technology transfer. In the context of seeking equitable benefit sharing for source countries according to obligations under the CBD, capacity building is widely recognised as an important benefit beyond the sharing of monetary benefits that arise from the utilisation of genetic resources. Berlinck et al [14] provide a valuable discourse of the values of these international collaborations as well as some of the hurdles, in an international collaboration with one developing country. The paradigm for benefit sharing by capacity building also stands true for international collaborations between developed countries. The case study of a partnership between the international pharmaceutical company AstraZeneca and an Australian university is a good example of the research funding, training, conservation benefits and infrastructure development that can arise from such partnerships [59].

**Figure 4.**
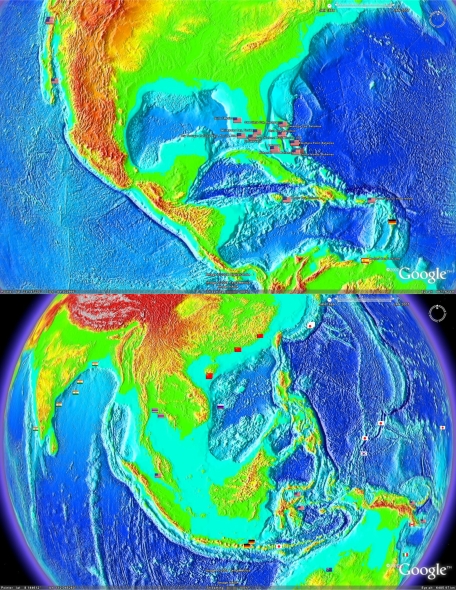
Demonstration of the ability to overlay more detailed information over the global surface. In this case, the data was downloaded from National Geophysical Data Centre, NOAA (Table 1).

While the aerial imagery used by Google Earth™ provides excellent topographical detail of features on land, and above water or in shallow water, underwater topographical features are missing from the picture. This can be rectified by overlaying a more detailed topographical dataset, such as that available from the National Geophysical Data Centre (National Ocean and Atmosphere Administration, USA) and shown with portions of the earth and a corresponding subset of the geobibliography in Figure 4. This provides an even more detailed visualisation of the locations where previous microbial marine natural product research has been conducted as well as that for regions yet to be explore.

**Figure 5.**
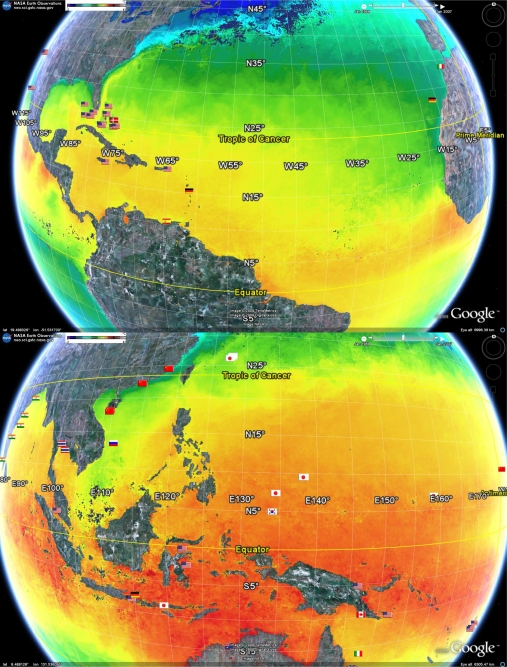
Distribution of research efforts related to sea surface temperature (SST). The depicted SST’s are the composites from December 1 2007 to January 1 2008 obtained from the NASA Earth Observatory program using data from both the AQUA and MODIS satellites. Increasing redness indicates increased SST

Figure 5 shows a one month snapshot of Sea Surface Temperatures (SST) overlaying the geobibliography. The temperature data used is a composite of SSTs recorded during the period December 1 2007 to January 1 2008. The SST dataset used here and displayed in Figure 5 is but one example of multiple datasets from many different time periods that can be downloaded form the NASA Earth Observatory (http://earthobservatory.nasa.gov/). It is known that SSTs can change at a range of time scales, from daily to longer term. Annual and decadal changes in patterns of SST can be influenced by changes in global phenomena such as the El Nino Southern Oscillation that occurs in the Pacific [67]. As a result, warm water can oscillate between the northern and southern hemispheres in the different seasons. Thus, access to alternative datasets would enable a range of alternate analyses of the geobibliography, such as examining the prevailing sea surface temperature regime that existed when a particular organism was collected for a reported publication.

Nevertheless, the snap-shot illustrated in Figure 5 effectively highlights the environmental complexity of the tropics. For the one month period depicted, the sea surface was quite cool along the tropical coastlines of Southern China and Vietnam (south of the Tropic of Cancer), while the ocean at similar latitudes in the same time period are warm. As a comparative example, note the significant warm pool centred within the Indonesian archipelago. Similarly, the north-west tropical coast of Africa is relatively cool compared to the Caribbean which was warm during the same period. Figure 5 also shows the tongue of cool water that can extend along the equator from the north-west coast of the South American continent. This picture shows that many tropical locations studied in the geobibliography spend at least some time in cool water, while areas outside the Tropics of Cancer and Capricorn spend at least some time in warm water. The ability to visualise and include environmental data, such as SST and productivity, can be extremely useful in biodiscovery research. This is because it may help explain, and therefore potentially predict and strategically target, patterns in bioactivity.

**Figure 6.**
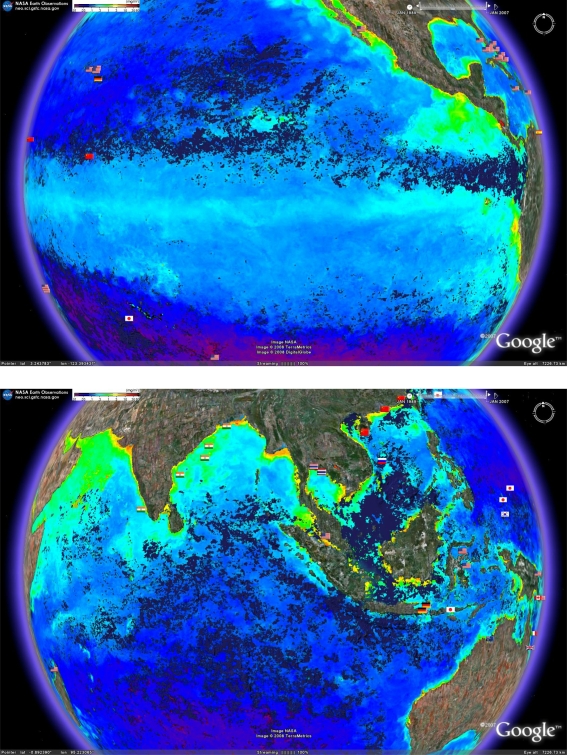
Proximity to areas of high productivity as measured by surface chlorophyll concentrations using composite date from the AQUA and MODIS satellites and for the time period of December 1 2007 to January 1 2008. Data can be downloaded from the NASA Earth Observatory (http://neo.sci.gsfc.nasa.gov/Search.html). Increased yellow intensity indicates increased chlorophyll concentration.

Another environmental variable which can fluctuate strongly across the tropics and therefore add a useful dimension to analyses for biodiscovery, is oceanic productivity. Chlorophyll concentrations in surface waters is widely regarded as a good proxy for oceanic productivity [11]. Figure 6 shows chlorophyll concentrations within surface waters during the same one month period as covered for SSTs in Figure 5. The resulting picture shows that during this month, the western coast of South and Central America were areas of high productivity, yet they have been the subject of virtually no reported marine microbial biodiscovery programs.

**Figure 7.**
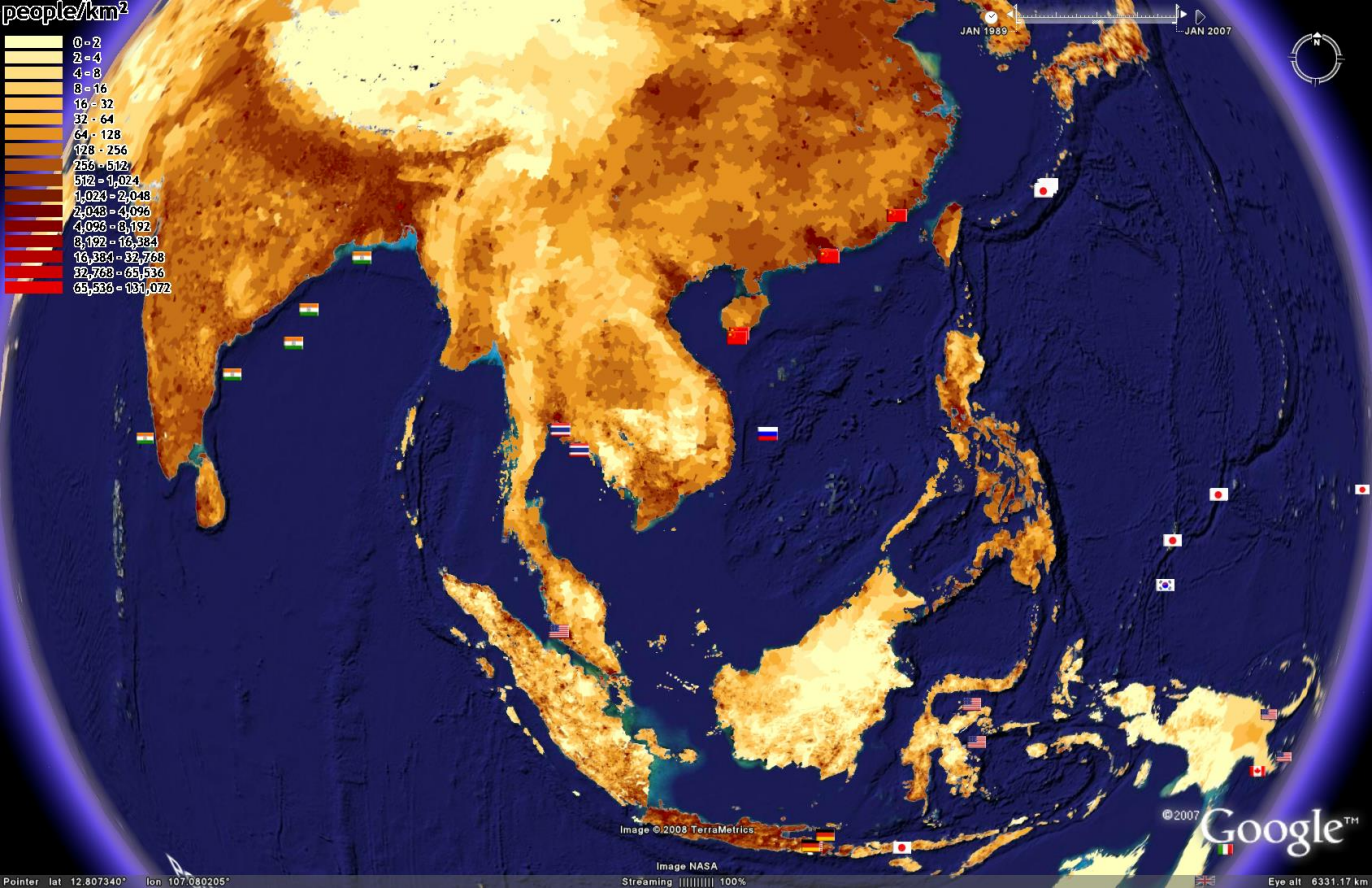
Geobibliography overlaid upon global population data (Center for International Earth Science Information Network and Centro Internacional de Agricultura Tropical, Columbia University, NY, USA) converted into a form able to to be used in Google Earth™ from http://bbs.keyhole.com/ubb/showflat.php/Cat/0/Number/92018/Main/90871

**Figure 8.**
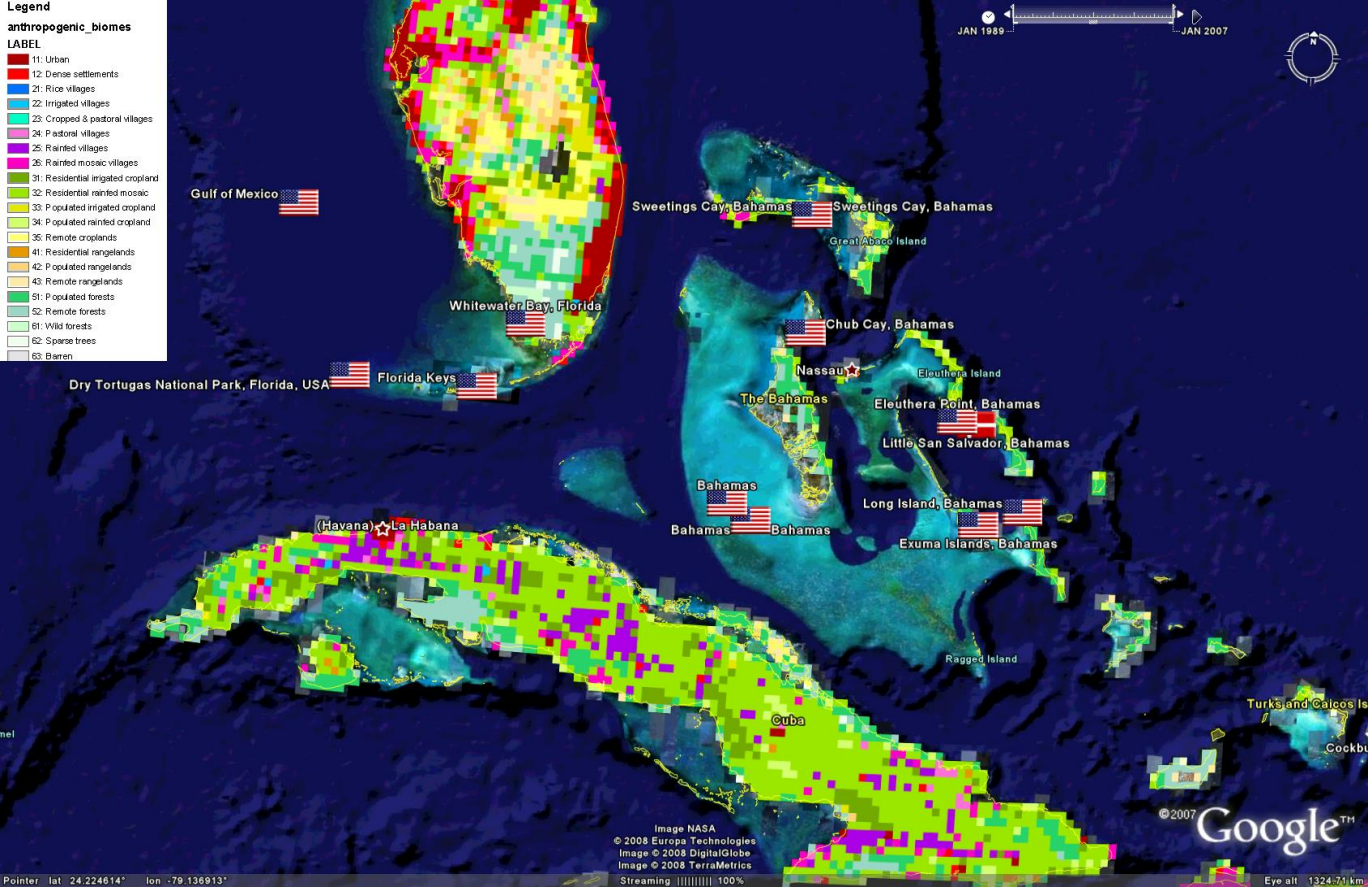

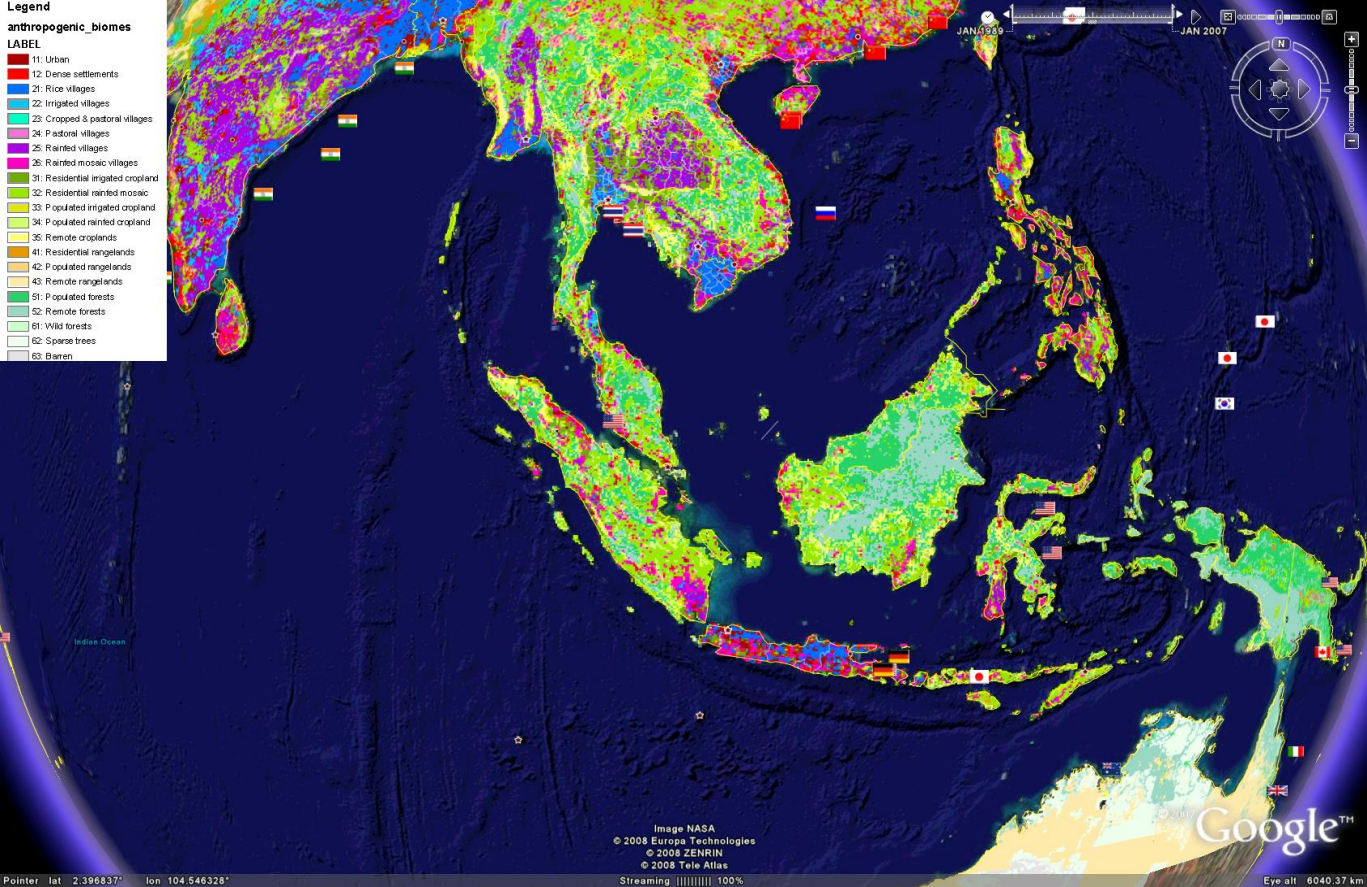
Anthropogenic biomes as described by Ellis and Ramankutty [25] in relation to tropical microbial marine biodiscovery efforts and the Google Earth™ file can be downloaded from www.eoearth.org/article/Anthropogenic_biome_maps

**Figure 9.**
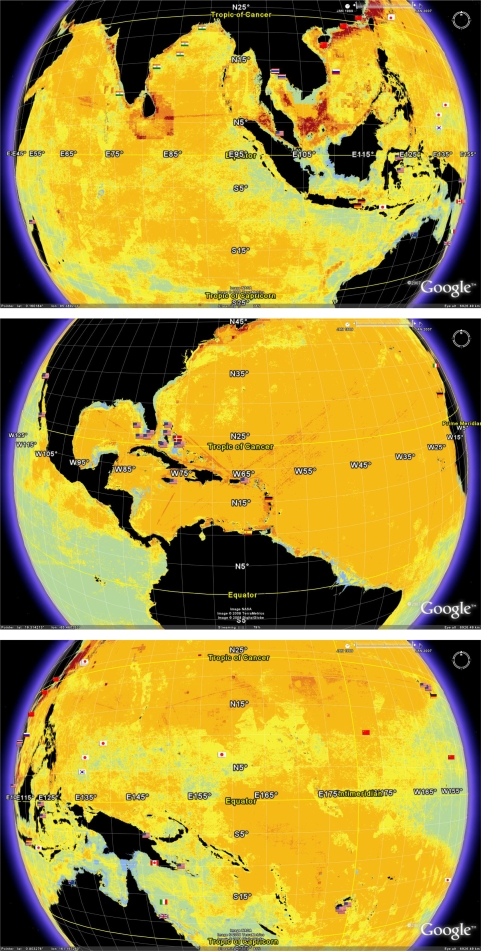
Location of tropical marine microbial natural product research relative to areas of human impacts upon the marine environment as described by Halpern et al [38]. Increasingly brown areas are areas of greater human impact with green areas being the least impacted. The author’s Google Earth™ file can be downloaded from http://www.nceas.ucsb.edu/GlobalMarine.

Figure 7 shows the location of tropical marine microbial biodiscovery research (in the geobibliography) overlaid with the location of global population. Some of the world’s most populated nations are located within the tropics and this can lead to significant pressures upon marine ecosystems through agricultural practices (eg sediment and agrichemical run-off), fishing, shipping and other human practices. Study of the impact of humanity upon nature has led the term *anthropocene* and the classification of the earth’s surface in terms of anthropogenic biomes [25], and measurements of resulting human impacts on the marine environment [38]. The existence of induced environmental stressors and their potential to elicit novel bioactivity, will be of interest to biodiscovery researchers. Figure 8 illustrates the geobibliography with respect to Ellis and Ramankutty’s biomes [25], while Figure 9 overlays the scale of human impacts on the marine environment as described by Halpern et al [38]. These figures indicate that few if any of the citations in the geobibliography have isolated marine microorganisms from marine ecosystems untouched by human activity.

**Figure 10.**
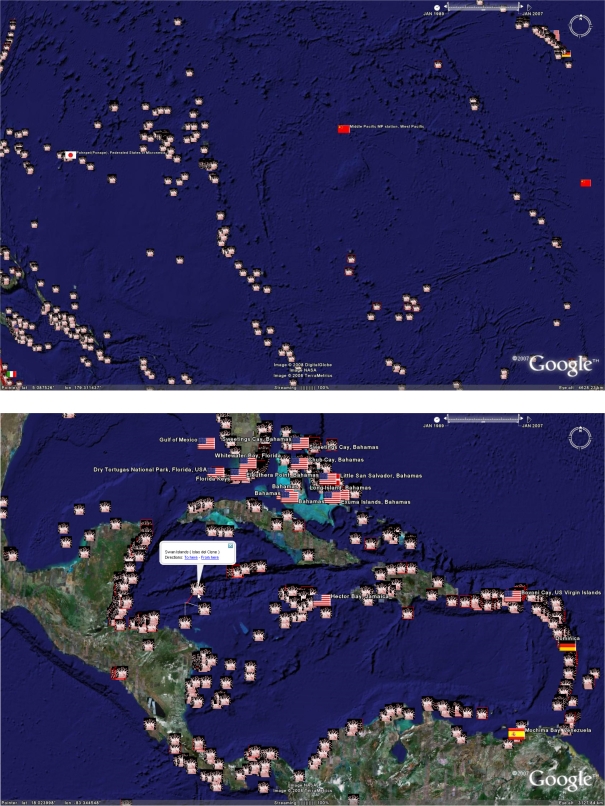
Locations of coal reefs within the Caribbean (upper panel) and the central Pacific (lower panel) relative to reported marine microbial natural product discoveries. Note that several of the reef icons possess a red edge which depicts their status as protected as recorded within the Reefbase maintained by the Worldfish Center.

Another striking and well celebrated feature of the tropical oceans is the prevalence of shallow water coral reefs. Figure 10 illustrates the geobibliography overlaid with the location of coral reefs recorded by Reefbase, an initiative supported by the United Nations Environment Program – World Conservation Monitoring Center and housed at the Worldfish Center in Penang, Malaysia. Reefs which are protected in some manner are indicated by a red edge to the icon. There are over 10,000 entries in this file, and the subsets shown in Figure 10 show that many of them have not been explored for microbial biodiscovery. These figures also highlight that only a small portion of the world’s reefs are protected, thus future biodiscovery programs on many of these reefs should proceed only with minimal impact collection methods. Microbial biodiscovery is ideal in this regard, as only minimal quantities of material are required to be taken from the field, and recollection is often unnecessary.

**Figure 11.**
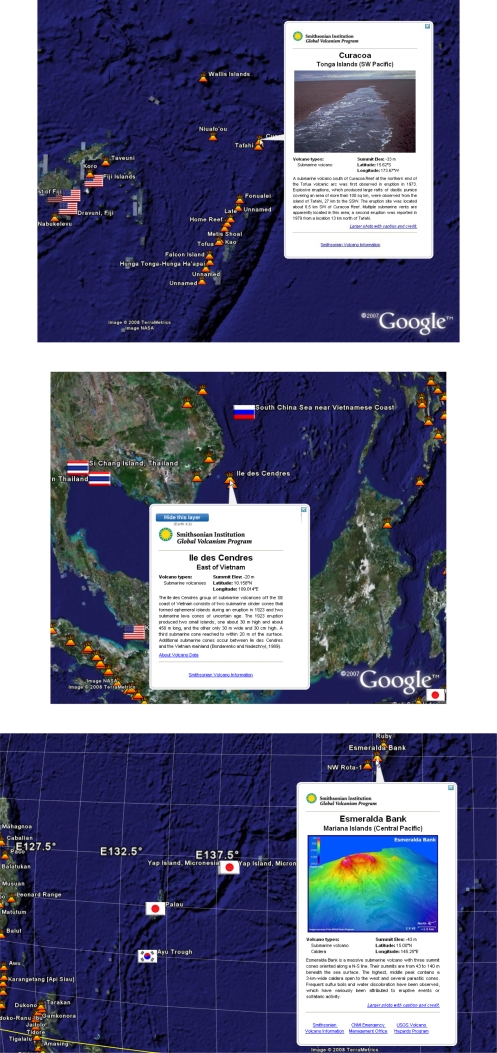
Proximity of marine microbial natural products research to undersea volcanoes. The examples shown are Curacao (upper panel) in the South West Pacific, Ill des Cendres south of Vietnam and the Esmerelda Bank (lower panel) in the Philippine Sea. All are within 50 meters of the sea surface and well within the reach of many marine scientific explorers.

Underwater volcanoes and sea mounts are other iconic marine ecosystems with biodiscovery potential. The geobibliography can be overlaid with the location of underwater volcanoes as recorded by the Smithsonian Institute’s volcano database. While many volcanoes occur in the ocean, especially along boundaries of tectonic plate activity, the majority are deep beneath the ocean surface and require specialised and expensive equipment and capability to enable sampling. A significant number however are within the upper reaches of the ocean and several examples are depicted within Figure 11. These and similar under-sea volcanoes extend to within 50m of the surface, and can be sampled using technology and instrumentation readily available to many within the marine scientific community.

**Figure 12.**
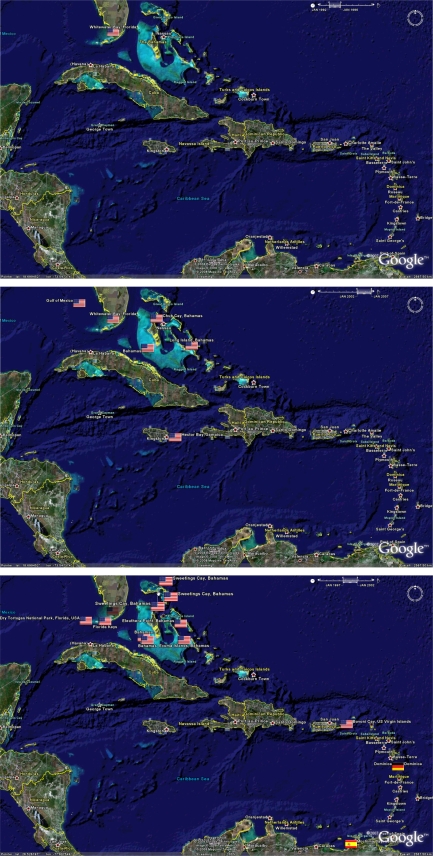
Research activity over time. The three panels below show the number of citations for Caribbean derived marine microbial natural products for the periods of a) January 1992–January 1995, b) January 1996 – January 1999, and c) January 2000 – January 2007.

Biodiscovery is an ancient science with a bright future, and many locations and ecosystems are yet to be visited. While the sub-field of marine microbial biodiscovery is by contrast relatively young, it has increased in activity over recent years as shown in Figure 12. It should be noted however that the year of publication is almost certainly a date after the collection and the reported research. In some cases, citations in the geobibliography report natural products isolated from microbes, which were collected and isolated several years before publication.

Geovisualisations such as those demonstrated in this paper, can facilitate hypothesis formulation by researchers, challenge accepted paradigms, and strategically select sampling sites and locations for future work. However, despite the power and potential of these tools, there remains an important pitfall in that they rely upon the quality of the available information. For example, many of the structures in the marine natural product literature cited in this project, are not yet available in facilities such as Pubchem. A remedy to this situation requires the natural products scientific community to proactively lodge new structures identified. The field of genetic and molecular science provides a useful model, whereby many journals require scientists to have lodged sequences in the publicly accessible database Genbank before papers can be published. Similarly, the geographic location of collections is not always provided by the authors of marine microbial natural product literature, and these papers have been excluded from the current study. As technologies for the ease and accuracy of measuring location improve (eg through the use of inexpensive hand-held GPS systems), then it could be expected that this deficiency will be redressed over time. A further incentive may come in the future with the adoption of source location disclosure and proof of legal provenance, as outlined earlier in the discussion on CBD compliance.

While this project focused on marine microbial natural products research, this compilation could readily be extended to all of the world’s biodiscovery efforts encompassing all marine ecosystems, terrestrial locations and macroscopic organisms. The objective of this paper was to highlight the power of current webGIS tools in data visualisation for complex analysis and interpretation. The datasets and tools used in this paper are readily available, thus enabling universal desktop global exploration. The resource costs involved are modest, and include a computer, an internet connection, the time to transfer data, and if applicable and desired, subscriptions to specialist databases and in this case, literature services. While the foundation used in this project is Google Earth™, it is but one example of a range of available options to underpin such visualisations. Similarly, while our focus has been the literature on marine microbial natural products research, this too should be regarded as an example of a geo-referenced dataset that can be generated in KML format and overlaid with the powerful visual functionality of software such as Google Earth™.

Globalisation is a concept that can be incorporated into scientific thinking and planning and this paper describes a tool and approach that can contribute to the rational analysis of the wealth of information provided in the scientific literature and other data sources. The reader is now invited to use our compilation and follow the principles we have outlined to generate their own geobibliographies, to explore the extensive potential of this new geo-visualisation genre.

## Figures and Tables

**Table 1.** Data sets downloaded for incorporation into the present study, and their source.

Downloaded Data Sets (KML format unless otherwise indicated)	Source
Boundaries of Exclusive Economic Zones	Vlaams Instituut voor de Zee [6] from http://bbs.keyhole.com/ubb/showthreaded.php/Cat/0/Number/693959/page/vc/vc/1
Bathymetric imagery	National Geophysical Data Centre, NOAA
Millennium Development Goals	Millennium Development Goal Monitor (www.mdgmonitor.org) gives global socioeconomic information
Sea Surface Temperature composites for the period 1 December 2007 – 1 January 2008.	NASA Earth Observatory program (www.neo.sci.gsfc.nasa.gov)
Chlorophyll composites for the period 1 December 2007 – 1 January 2008.	NASA Earth Observatory program, data from AQUA and MODIS satellites Available at www.neo.sci.gsfc.nasa.gov
Global population data	Center for International Earth Science Information Network and Centro Internacional de Agricultura Tropical, Colombia University, NY, USA. Google Earth™ version downloadable from www.bbs.keyhole.com/ubb/showflat.php/Cat/0/Number/92018/Main/90871
Anthropogenic biomes.	Described by Ellis and Ramankutty [25] with KML file at www.eoearth.org/article/Anthropogenic_biome_maps
Impact of human activities	Google Earth™ file supporting Halpern et al [38] downloadable from www.nceas.ucsb.edu/GlobalMarine
Location of coral reefs. This data needed tobe converted to KML format.	Worldfish Center’s Reefbase (www.reefbase.org)
Location of undersea volcanoes.	Smithsonian Institution Global Volcanism Program. Available for download at www.volcano.si.edu
